# Use, knowledge, and perception of the scientific contribution of Sci-Hub in medical students: Study in six countries in Latin America

**DOI:** 10.1371/journal.pone.0185673

**Published:** 2017-10-05

**Authors:** Christian R. Mejia, Mario J. Valladares-Garrido, Armando Miñan-Tapia, Felipe T. Serrano, Liz E. Tobler-Gómez, William Pereda-Castro, Cynthia R. Mendoza-Flores, Maria Y. Mundaca-Manay, Danai Valladares-Garrido

**Affiliations:** 1 Escuela de Medicina Humana, Universidad Continental, Huancayo, Perú; 2 Escuela de Postgrado, Universidad Privada Antenor Orrego, Trujillo, Perú; 3 Unidad de Investigación en Enfermedades Emergentes y Cambio Climático (Emerge), Universidad Peruana Cayetano Heredia, Lima, Perú; 4 Universidad Privada de Tacna, Tacna, Perú; 5 Grupo de investigación ACEMED-UPTC, Universidad Pedagógica y Tecnológica de Colombia-UPTC, Tunja, Colombia; 6 SOCIEMAP, Universidad Nacional de la Amazonía Peruana, Loreto, Perú; 7 SOCEMUNT, Universidad Nacional de Trujillo, Trujillo, Perú; 8 ASOCEM-UANCV, Universidad Andina Néstor Cáceres Velásquez, Juliaca, Perú; 9 Facultad de Medicina Humana, Universidad Nacional Pedro Ruiz Gallo, Lambayeque, Perú; Humboldt-Universität zu Berlin, GERMANY

## Abstract

**Introduction:**

Sci-Hub is a useful web portal for people working in science as it provides access to millions of free scientific articles. Satisfaction and usage should be explored in the Latino student population. The objective of this study was to evaluate the use, knowledge, and perception of the scientific contribution of Sci-Hub in medical students from Latin America.

**Methodology:**

A multicenter, observational, analytical study was conducted in 6632 medical students from 6 countries in Latin America. We surveyed from a previously validated instrument, delving into knowledge, monthly average usage, satisfaction level, and perception of the scientific contributions provided by Sci-Hub. Frequencies and percentages are described, and generalized linear models were used to establish statistical associations.

**Results:**

Only 19.2% of study participants knew of Sci-Hub and its function, while the median use was twice a month. 29.9% of Sci-Hub-aware participants claimed they always find the desired scientific information in their Sci-Hub search; 62.5% of participants affirmed that Sci-Hub contributes to scientific investigation; only 2.2% reported that Sci-Hub does not contribute to science.

**Conclusion:**

The majority of Latino students are not aware of Sci-Hub.

## Introduction

Sci-Hub is a website that contains about 50 million papers from scientific journals, providing free access to researchers worldwide [1,2]. 200,000 articles are downloaded daily from this database, without any distinction between developing and developed countries, where the main source, Elsevier, accounts for 50% of the downloads [1]. The Latino scientific community is well aware of this resource; in Peru, from September 2015 to February 2016, just over 370,000 papers were downloaded [2].

Research utilizing Sci-Hub is scarce globally, as regions of Latin America have not demonstrated its usage, particularly among medical professionals. There have been some small reports that have discussed the scientific ethical conduct of this website due to the illegality of its service, while others have defended this site based on the high costs of scientific publications, leading many scientists to elect to obtain papers illegally [3–7].

The knowledge, use and perception of Sci-Hub and the use of Sci-Hub among medical students have not yet been explored. The purpose of this study was to determine the use, knowledge and perception of the scientific contribution offered by Sci-Hub in Latin American medical students.

## Methodology

### Study design

A cross-sectional, analytic, observational multicenter study was conducted.

### Location and time

This study was conducted from February-June 2016, in medical students from 6 Latin American countries.

### Sampling

Stratified random sampling was performed (utilizing each year of study as a stratum). The sample size was calculated with a power of 80%, with 95% statistical significance for an infinite population. The determined minimal sample size for each site was 289 students, and 10% was added to that value to account for possible losses, obtaining a final value of 318 medical students at each site. For randomization, a stratified sample proportional to each year of study was used and each sample was proportional to each site collection size. For the selection of participants, each delegate entered the classroom with the highest credit in each academic year, and students who were sitting in an odd numbered location in each of the rows were selected until the required sample size for each year of study was fulfilled. In the universities with equal or smaller student populations than the minimum sample size (3 universities), a census-type sampling was conducted.

Medical students who were enrolled in the class period 2016-I were included, and participation was voluntary. Students who were in their medical internship period or who did not answer the variables of interest of the questionnaire were excluded.

### Procedures

A previously validated questionnaire designed by authors was used (the questionnaire was reviewed by experts and underwent a pilot study to evaluate the relevance and understanding of the questions). The questionnaire consisted of questions divided into two sections: (1) socio-educational data and (2) knowledge, monthly average usage, satisfaction level, and perception of scientific contributions of Sci-Hub. (S1 File)

The socio-educational variables included sex, age, year of study, knowledge of the English and Portuguese language, previous career, as well as membership in study group (defined as groups dedicated only to academic activities), scientific societies of medical students (SSMS), or research groups.

The knowledge about the use of Sci-Hub was measured in two aspects: awareness of Sci-Hub and function of Sci-Hub. The usage was explored through the average number of times of use per month. The level of satisfaction was measured with tiers of search success: always, sometimes, or never find what you want. Finally, a question was asked to explore the perception of whether Sci-Hub contributed to research with three response alternatives: agree, indifferent, disagree.

A convocation of official social networks of FELSOCEM, *“Federación Latinoamericana de Sociedades Científicas de Estudiantes de Medicina”*, met and e-mail invitations were sent directed to delegates and presidents of every scientific society during the months of September-December 2015. From this, the participation and commitment of universities of 6 countries of Latin America (Peru, Bolivia, Colombia, Chile, Paraguay, and Argentina) were obtained.

Later, participants coordinated with their respective managers of the universities, distributing the instrument and offering advice and training to execute the study across virtual meetings. Every coordinator requested permission of the headquarters faculty by sending the protocol and document of approval of the committee of ethics for the researcher responsible for the study. Deadlines were established to compile and tabulate the data following the chronogram of the study.

### Ethics

This project was approved by the Committee of Ethics of the “Hospital Nacional Docente Madre Niño” supported by the National Institute of Health of Peru. The surveys were auto-administered and anonymous, and the privacy of the participants was respected through the use of digital codes. The importance and intention of the study was explained beforehand to participants.

### Statistical analysis

Each office manager made a digital database in Microsoft Excel, with constant quality control by the principal investigator of the study.

Statistical analysis was performed with Stata program v.11.1 (StataCorp LP, College Station, TX, USA).

For the descriptive analysis of numerical variables, normality assumptions were evaluated using the Shapiro-Wilk test. According to the test, the best measure of central tendency and dispersion were described through categorical variables described in frequencies and percentages.

Bivariate and multivariable analysis was performed using knowledge of Sci-Hub as the dependent variable. Reported p values were obtained by Chi-square test (set by Fisher's exact test) for the association between knowledge of Sci-Hub and other variables. For bivariate and multivariate statistics, generalized linear models (GLM acronym in English) using Poisson family, link function log, and robust models were used, and respondents were considered as clusters at each venue (under the assumption that the groups differ according to their teachings, study methodologies and curricula). A value of p <0.05 was considered as significant.

## Results

Of 6632 medical students, 51.5% (3415) were women; the median age was 21 years (interquartile range: 19–23 years) (S1 Fig); the country that contributed the most respondents was Peru (60.0%); and 50.4% (3346) of responses came from several private universities. **(cf. Table 1)**

**Table 1:** Distribution and demographics of medical students from 6 Latin American countries.

Variable	N	%
**Sex**		
Female	3415	51.5
Male	3217	48.5
Age (years)*	21	19–23
**Country of residence**		
Peru	3982	60.0
Paraguay	885	13.3
Bolivia	642	9.7
Argentina	569	8.6
Colombia	318	4.8
Chile	236	3.6
**Type of university**		
Public	3286	49.6
Private	3346	50.4

*Median and interquartile range.

Of those who responded, 5359 (80.8%) were unaware of Sci-Hub. The median frequency of usage of Sci-Hub was 2 days per month (interquartile range: 1–4); 369 (29.9%) of respondents reported they always find what they were searching for; and 783 (62.5%) of respondents believe that Sci-Hub contributes to research. **(cf. Table 2)**

**Table 2:** Usage of Sci-Hub amongst medical students from 6 Latin American countries.

Variable	N	%
**Awareness of Sci-Hub?**		
No	5359	80.8
Yes	1273	19.2
Average use (days per month)*	2	1–4
**Level of satisfaction**		
I always find what I want	369	29.9
Sometimes I find what I want	841	68.3
I never find what I want	22	1.8
**Positive contribution to research**		
Yes	783	62.5
Indifferent	442	35.3
No	27	2.2

*Median y interquartile range.

**Fig 1:**
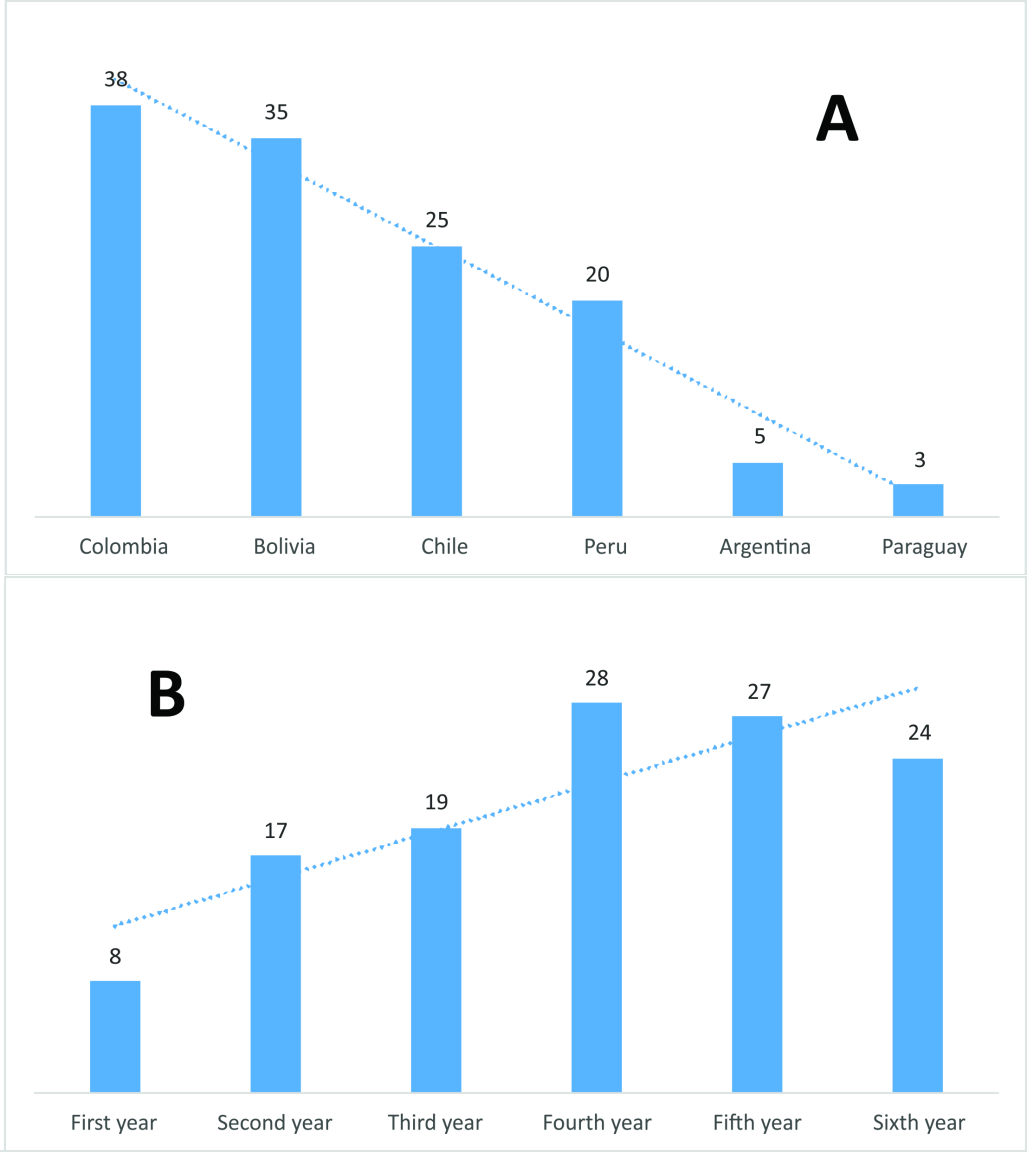
(A) Percentage of students with knowledge of Sci-Hub by country and (B) by year of study.

As shown in (**Fig 1A)**, the highest percentage of awareness of Sci-Hub by country of residence of students was in Colombia (38%) followed by Bolivia (35%). Meanwhile, (**Fig 1B)** shows that knowledge of Sci-Hub increases in proportion to the ascension of the year of study in medical school.

The bivariate analysis demonstrated an association between awareness of Sci-Hub and sex (p <0.001), age (p = 0.015), academic year (p = 0.017), membership in a Scientific Society of Medical Students (SSMS) (p = 0.036), completion of a curricular project (p <0.001) and an extracurricular project (p <0.001). **(cf. Table 3)**

**Table 3:** Bivariate analysis of knowledge of Sci-Hub among medical students from 6 Latin American countries.

Variable	Known Sci-Hub N (%)		cPR (CI95%)	*p* value
	Yes	No		
Sex				
Female	563(44.2)	2852(53.2)	**0.75(0.65–0.86)**	**<0.001**
Male	710(55.8)	2507(46.8)		
**Age** (years)*	21(20–23)	21(19–23)	**1.07(1.01–1.12)**	**0.015**
**Academic stage**				
Clinical sciences	880(70.3)	2562(50.7)	**1.97(1.13–3.44)**	**0.017**
Basic sciences	372(29.7)	2494(49.3)		
**University**				
Private	627(49.3)	2659(49.6)	0.99(0.46–2.13)	0.976
Public	646(50.7)	2700(50.4)		
**Group membership**				
SSMS	277(21.8)	726(13.6)	**1.56(1.02–2.37)**	**0.036**
Study group	410(32.2)	2298(42.9)	0.69(0.47–1.02)	0.062
Any group	462(36.3)	2180(40.7)	0.86(0.54–1.37)	0.526
**Completed Project**				
Curricular	1008(79.4)	2704(50.7)	**2.99(1.92–4.66)**	**<0.001**
Extracurricular	372(29.3)	771(14.6)	**1.96(1.43–2.68)**	**<0.001**

cPR (crude prevalence ratio), CI95% (confidence interval 95%) and p value obtained with generalized linear models with Poisson family, log link function and robust models. SSMS: Scientific Society of Medical Students.

*Median and interquartile range.

The multivariate analysis revealed an association between a higher frequency of awareness of Sci-Hub and membership in a SSMS (aPR:1.44; 95% CI:1.19–1.75; p value<0.001), in addition to completion of a curricular project (aPR:1.43; 95% CI:1.06–1.94; p value:0.020) or an extracurricular project (aPR:2.48;95% CI:1.69–3.65; value p<0.001). It was also found that women had a lower frequency of knowledge of Sci-Hub (aPR:0.78;95% CI:0.70–0.87; p value<0.001), and even when adjusting for age, this trend prevailed in clinical science courses and by country of origin. **(cf. Table 4)**

**Table 4:** Multivariate analysis of knowledge about Sci-Hub according to socio-educational variables among medical students from 6 Latin American countries.

Variable	aPR (CI95%)	*p value*
**Female**	**0.78(0.70–0.87)**	**<0.001**
**Age (years)**	1.03(0.98–1.07)	0.213
**Clinical courses**	1.36(0.88–2.08)	0.165
SSMS* **member**	**1.44(1.19–1.75)**	**<0.001**
**Completed Project**		
Curricular	**1.43(1.06–1.94)**	**0.020**
Extracurricular	**2.48(1.69–3.65)**	**<0.001**

aPR (Adjusted prevalence ratio), CI95% (confidence interval 95%) and *p value* obtained with generalized linear models with Poisson family, log link function, robust models and using the country as an adjustment (cluster).

*SSMS: Scientific society of medical students.

## Discussion

Four out of every five students were unaware of Sci-Hub despite it being a very valuable resource for obtaining scientific literature, and in recent years, being an important resource for conducting research. This low frequency may be because Sci-Hub is currently not considered legal [5] and its URL is constantly changing in response to legal repression [8]. Its lack of a constant URL may be impeding widespread usage and may prevent researchers from sharing this website, as it is possible that when someone tries to use its latest URL, it has already been modified, resulting in additional time spent finding the new URL and domain. Legal research databases and programs used for the search of scientific articles have reported high rates of knowledge and use [9,10], due to its fixed URL, which allows information about their existence to be shared with many more researchers, therefore increasing its use.

Almost all respondents aware of Sci-Hub reported that they either always or sometimes find what they need in Sci-Hub, keeping into consideration that not all scientific literature is always available in one place, and those with very old/new systems or with extreme security Web protection are unable to download from the server. Additionally, Sci-Hub is not only a resource that contributes to research, but also aids in generating additional research, as described by McNutt [4], Resnik [11] and Priego [6]. Researchers and students use Sci-Hub as an alternative means for access to various scientific articles and updated information for the development of their daily clinical practice, although its usage is considered an ethical dilemma [12].

Fewer women than men had knowledge of Sci-Hub (44.2% of female vs. 55.8% of male respondents). This could be explained by the fact that despite the great progress that women have made in recent years in the medical sciences [13, 14], multiple studies have shown that there is still a predominance of men in scientific research [15–17]. Additionally, it has been shown that only 29.3% [15] to 34% [16] to 36.7% [17] of first authors in research articles are women, which is considerably low. Fewer female scientists or gender discrimination may account for this large discrepancy between genders in awareness of Sci-Hub and perhaps other useful resources for scientific research.

Finally, it was found that of those who conduct research and belong to a Scientific Society of Medical Students (SSMS) or have completed a scientific curricular/extracurricular project have a higher rate of awareness of Sci-Hub. This suggests that those who conduct research employ this type of resource to gain access to scientific literature. Although there are currently no studies that affirm this association, students that belong to SSMS often face the same obstacles of researchers, as they are often involved in research and publish scientific articles [18–19]. Therefore, these students also require programs or portals like Sci-Hub for access to scientific information. In a report on requests for downloads on Sci-Hub [1], it was found that these requests were mainly made by members of the scientific community, with Elsevier medical publishing being the most accessed.

This study had limitations, such as selection bias, since delegates distributed surveys to students in courses with higher credits. However, it is known that some students do not attend the same classes, since in many universities attendance is not compulsory in order to reduce the number of students for randomization. Another important limitation is the limited literature available in journals on the use and knowledge of Sci-Hub by health professionals and medical students predominantly from a Latin American country; however the latter was not a significant issue due to the sampling rate used and the large sample size. Despite these limitations, this study provides the basis for future reports, which can include more variables to find a relationship to knowledge of Sci-Hub.

## Conclusions

Latin American students who are aware of Sci-Hub often find what they want using this database and the average use of this site is twice a month. Membership in a SSMS and participation in a curricular or extracurricular project are characteristics associated to knowing this research portal. More than half of the students who were aware of Sci-Hub agreed that Sci-Hub positively contributed to research. However, the vast majority of students are unaware of this resource, despite it being a very useful tool, as it offers free access to a large number of research articles in scientific journals. Despite the contested ethics behind this site, this portal still provides an alternative for access to valuable, updated scientific information for the student population.

## Supporting information

S1 File Questionnaire. (PDF)

S1 Fig Age distribution. Age distribution of medical students from six Latin American countries.
(TIF)

## References

[1] Bohannon J. Who’s downloading pirated papers? Everyone. Science [Internet]. April 28, 2016 [cited May 24, 2016]; Available in: http://www.sciencemag.org/news/2016/04/whos-downloading-pirated-papers-everyone 10.1126/science.352.6285.508 27126020

[2] Gracias a Sci-Hub dejamos de mendigar papers—expresiongenetica | Blogs | El Comercio Peru [Internet]. [cited May 24, 2016]. Available in: http://elcomercio.pe/blog/expresiongenetica/2016/05/gracias-a-sci-hub-dejamos-de-mendigar-papers

[3] Bohannon J. The frustrated science student behind Sci-Hub. Science. 2016; 352(6285):511 doi: 10.1126/science.352.6285.511 27126021 10.1126/science.352.6285.511

[4] McNutt M. My love-hate of Sci-Hub. Science. 2016; 352(6285):497 doi: 10.1126/science.aaf9419 27126012 10.1126/science.aaf9419

[5] Russell C, Sanchez E. Sci-Hub unmasked Piracy, information policy, and your library. Coll Res Libr News. 2016; 77(3):122–5.

[6] Signal, Not Solution: Notes on Why Sci-Hub Is Not Opening Access | The Comics Grid (Blog) | Ernesto Priego [Internet]. [cited May 24, 2016]. Available in: http://blog.comicsgrid.com/2016/02/sci-hub-not-open-access/

[7] Fuchs C, Sandoval M. The Diamond Model of Open Access Publishing: Why Policy Makers, Scholars, Universities, Libraries, Labour Unions and the Publishing World Need to Take Non-Commercial, Non-Profit Open Access Serious. TripleC. 2013; 11(2):428–43.

[8] Faust JS. Sci-Hub. Ann Emerg Med. 2016; 68(1):A15–7.

[9] Caballero AD, Martínez GR, Martínez FG. Percepción del desempeño en la búsqueda de información en bases de datos bibliográficas de los estudiantes de estomatología. Caso de estudio. ACIMED. 2010; 21(1):0–0.

[10] Ospina EG, Herault LR, Cardona AF. The use of bibliographic databases by Spanish-speaking Latin American biomedical researchers: a cross-sectional study. Rev Panam Salud Pública. 2005; 17(4):230–6. 15969974 10.1590/s1020-49892005000400003

[11] Resnick B. Why one woman stole 50 million academic papers—and made them all free to read [Internet]. Vox. 2016 [cited July 23, 2016]. Available in: http://www.vox.com/2016/2/17/11024334/sci-hub-free-academic-papers

[12] Bendezú-Quispe G, Nieto-Gutiérrez W, Pacheco-Mendoza J, Taype-Rondan A. Sci-Hub and medical practice: an ethical dilemma in Peru. Lancet Glob Health. 2016; 4(9):e608 doi: 10.1016/S2214-109X(16)30188-7 27539805 10.1016/S2214-109X(16)30188-7

[13] Ramakrishnan A, Sambuco D, Jagsi R. Women’s Participation in the Medical Profession: Insights from Experiences in Japan, Scandinavia, Russia, and Eastern Europe. J Womens Health. 2014; 23(11):927–34. 10.1089/jwh.2014.4736 PMC4235590 25320867

[14] Okoshi K, Nomura K, Fukami K, Tomizawa Y, Kobayashi K, Kinoshita K, et al Gender Inequality in Career Advancement for Females in Japanese Academic Surgery. Tohoku J Exp Med. 2014; 234(3):221–7. 25355369 10.1620/tjem.234.221

[15] Jagsi R, Guancial EA, Worobey CC, Henault LE, Chang Y, Starr R, et al. The «Gender Gap» in Authorship of Academic Medical Literature—A 35-Year Perspective. N Engl J Med. 2006; 355(3):281–7. doi: 10.1056/NEJMsa053910 16855268 10.1056/NEJMsa053910

[16] Filardo G, Graca B da, Sass DM, Pollock BD, Smith EB, Martinez MA-M. Trends and comparison of female first authorship in high impact medical journals: observational study (1994–2014). BMJ. 2016; 352:i847 doi: 10.1136/bmj.i847 26935100 10.1136/bmj.i847 PMC4775869

[17] Sidhu R, Rajashekhar P, Lavin VL, Parry J, Attwood J, Holdcroft A, et al The gender imbalance in academic medicine: a study of female authorship in the United Kingdom. J R Soc Med. 2009; 102(8):337–42. doi: 10.1258/jrsm.2009.080378 19679736 10.1258/jrsm.2009.080378 PMC2726808

[18] Taype-Rondán A, Huaccho-Rojas J, Guzmán L. Sociedades científicas de estudiantes de medicina en el Perú: situación actual y perspectivas futuras. CIMEL. 2011; 16(2):90–95.

[19] Taype-Rondán A, Bazán-Ruiz S, Valladares-Garrido D. Producción científica de las sociedades científicas de estudiantes de medicina del Perú, 2002–2012. CIMEL. 2013; 18(1):23–29

