# Supplementary material for: Use, knowledge, and perception of the scientific contribution of Sci-Hub in medical students: Study in six countries in Latin America
Source: PLoS One. 2017 Oct 5;12(10):e0185673. doi: 10.1371/journal.pone.0185673 (PMC5628842; doi:10.1371/journal.pone.0185673)
Supplement: S1 File — (PDF) [file pone.0185673.s001.pdf]

APPENDIX 1: Questionnaire

QUESTIONNAIRE

Use, knowledge, and perception of the scientific contribution of Sci-Hub in medical students: study in six countries in Latin America.

Dear Student: Your participation in this research is voluntary, with the filling of the survey understand that providing consent to participate. The data provided are confidential and anonymous. The results will be published as aggregated data in a scientific journal.

Thank you very much for your help

Respondent Date: ____ / ____ / 2016 Country/City: _____________________

University: ________________________________, This university is: Public ( ) Private ( )

CHARACTERISTICS OF RESPONDENT

1. **Sex:** Male ( ) Female ()
2. **Age:** _____ (years old)
3. **You are conducting currently on:** Cycle: ______ (eg 1st, 2nd, 3rd) Year: ______ (eg 1st, 2nd)
4. **Previously You studied another career:** No ( ) Yes ( ), if the answer was yes: Which career: __________
5. **It belongs to (you can check more than one):** Study Groups ( ) Scientific Society (SOCEM) ( ) Research Group ( )
6. **Have you completed any research projects during the courses of the Career? (in classes)** No ( ) Yes ( )
7. **Have you completed any extracurricular research projects? (by your own)** No ( ) Yes ( )

Sci-Hub

1. **Do you know what is Sci-Hub and what it does?** No ( ) Yes ( )
2. **Have you used Sci-Hub?** No ( ) Yes ( ), **If your answer was yes, tell how many times you use on average per month:** _______
3. **Tell your satisfaction level when using Sci Hub:** Always find what I want ( ) - I find sometimes ( ) - never find what I want ( )
4. **Do you think Sci-Hub contribute to research?** Agree ( ) - Indifferent ( ) - Disagree ( )

The survey is finished, thank you very much for your cooperation.
