# Supplementary figures and images for: Use, knowledge, and perception of the scientific contribution of Sci-Hub in medical students: Study in six countries in Latin America
Source: PLoS One. 2017 Oct 5;12(10):e0185673. doi: 10.1371/journal.pone.0185673 (PMC5628842; doi:10.1371/journal.pone.0185673)

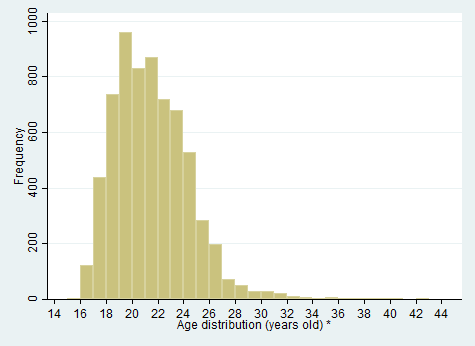

Supplement: S1 Fig — Age distribution of medical students from six Latin American countries. (TIF) [file pone.0185673.s002.tif]
